# How Cognitive Reserve Could Protect from Dementia? An Analysis of Everyday Activities and Social Behaviors During Lifespan

**DOI:** 10.3390/brainsci15060652

**Published:** 2025-06-17

**Authors:** Francesca Morganti, Ilia Negri

**Affiliations:** 1 Department of Human and Social Sciences, University of Bergamo, 24129 Bergamo, Italy; 2 Centre for Healthy Longevity (CHL), Univesity of Bergamo, 24129 Bergamo, Italy; 3 Department of Economy Statistic and Finance, University of Calabria, 87036 Rende, Italy; ilia.negri@unical.it

**Keywords:** cognitive reserve, frailty in aging, dementia prevention, lifespan prevention, lifestyle risk reduction, stressors, social relationships

## Abstract

Background/Objectives: In the last decade, there has been a notable increase in the prevalence of cognitive decline among the elderly population. This phenomenon is further compounded by the concurrent rise in life expectancy, indicating a growing concern for the health and well-being of individuals in this demographic. Dementia has become a disease with a strong social impact, not exclusively limited to its health dimension. It is generally accepted that lifestyle factors and psychological attitudes toward life challenges may serve as protective mechanisms against pathological cognitive decline. The objective of this contribution is to evaluate the impact of lifestyle factors (e.g., physical activity, employment history, nutrition, technology use, etc.), stressors (e.g., illness, rare events, abandonments, home moving, etc.), and sociability (e.g., marriage, active friend network, children proximity, work relationships, etc.) at the onset of pathological cognitive frailty. Methods: In this study, a semi-structured interview was administered to 32 individuals over the age of 65 during their initial neuropsychological evaluation for suspected dementia. Results: Linear regressions with Mini Mental State Examination scores indicated that lifestyle and sociability factors offer a degree of protection against cognitive decline, while stressors were found to be unrelated to this phenomenon. Conclusions: The utilization of contemporary technologies, the possession of a driver’s license, and the maintenance of an active social network have been demonstrated to possess a high degree of predictive value with respect to cognitive reserve in the context of aging.

## 1. Introduction

The course of an individual’s life is characterized by progressive transformations, with the passage of time resulting in observable changes within the body. These changes extend beyond the physical realm to encompass the social and emotional spheres. The deterioration of the older individual’s social role, accompanied by a decline in integrity and self-esteem, is often a gradual and insidious process. It can be argued that an individual’s maladjustment is not solely a consequence of aging, but, rather, is largely due to emotional factors related to social integration. This phenomenon is often associated with the onset of anxiety, social isolation, and frustration in elderly individuals. Prolonged exposure to these feelings can have a detrimental effect on cognitive function, potentially leading to cognitive decline. The emotional dimension of the aging process is a subject of ongoing research, focusing on the individual’s continuous examination of the emotional significance of life events [1]. A particularly salient occurrence is the transition from the workforce to retirement. The decision to exit the workforce can be viewed as either favorable or unfavorable. In many cases, the influence of one’s occupation extends beyond the professional sphere, affecting personal relationships and leisure activities. The sudden change in lifestyle can have positive and negative outcomes for the individual. The specific consequences of this phenomenon are contingent upon the individual’s response. At this juncture, the navigation of the subject’s growth can be facilitated by the identification of new resources. The identification of these resources is contingent upon the subject’s capacity and willingness to identify novel avenues for growth.

The inevitability of universal and irreversible changes associated with aging does not inherently render the individual impaired. Indeed, the process of aging is accompanied, to a certain extent, by a gradual decline in physical strength and resilience. The term “frailty” in the context of aging can be defined as a reduction in an individual’s ability to effectively cope with environmental challenges. As a result, the individual may become more susceptible to environmental factors and demonstrate an inability to perform certain tasks associated with daily living. The quality of frailty can vary considerably from one individual to another, depending on their inherited characteristics and lifestyle choices. It is imperative to acknowledge that the interplay between these two factors can precipitate a decline in the individual’s overall efficiency with advancing age, thereby engendering heightened vulnerability in the aged. Furthermore, the challenges associated with daily living frequently reduce the ability of older persons with cognitive frailty to function independently within their communities, often resulting in a significant degree of social isolation or the need for ongoing support from social institutions and caregivers.

The aim of this study is to delineate the boundaries of this intermediate state between aging, cognitive frailty, and dementia. Furthermore, it is imperative to ascertain the extent to which the decline of memory or other cognitive functions can be regarded as a normal phenomenon and the circumstances under which such decline is instead perceived as the initial phase of a dementia disease. This endeavor has been characterized as arduous [2]. The response to this question is not straightforward and will require further investigation, particularly given the current lack of clarity regarding the relationship between cognitive frailty and dementia [3,4,5]. The notion of frailty has historically been predominantly focused on the physical dimension [6]. Recent research has initiated a more thorough examination of the cognitive impairment associated with physical frailty, resulting in the conceptualization of cognitive frailty [7]. According to the work of Kelaiditi et al. [8], this is defined as the simultaneous presence of both physical frailty and cognitive impairment, without the presence of a concomitant neurological disease.

Cognitive frailty is regarded as a potential precursor of neurodegenerative processes with significant potential for reversal, making it an optimal target for early interventions. Conversely, the aging process may also be accompanied by a prevalent weakening of cognitive functions, including attention and memory, as well as an impairment in the acquisition and retention of new information or procedures [9]. Thus, age is the most consistent risk factor for cognitive decline worldwide, with dementia prevalence ranging from 2 to 11% in individuals under the age of 65. Concurrently, the onset of genuine cognitive deterioration signifies a critical juncture in the progression of the illness. Accordingly, the American Psychological Association [10] defines dementia as a progressive major neurocognitive syndrome disorder, which is characterized by neuropsychological impairments and a reduced ability to perform activities in everyday life. Dementia, in fact, is a chronic condition with a prolonged course; it is not the direct consequence of aging. The impact of aging and frailty on the onset of dementia remains a subject of scientific inquiry.

In recent years, a broad consensus has emerged regarding the necessity of identifying protective and risk factors for dementia [11]. This has been achieved through the utilization of an approach designated as the “Life Course” [12,13]. This theoretical model is employed to investigate the impacts of social and biological factors experienced by a cohort of individuals throughout their lifespan, as well as the subsequent influence of these factors in subsequent years [14,15]. The principal objective of this methodology is to furnish crucial insights into the aetiology of diseases and to develop and evaluate theoretical models that aim to identify significant correlations between life experiences and the onset of diseases in later life.

Furthermore, the correlations identified between the assumption of a specific lifestyle and the multiplicity of risk factors that contribute, in the context of human existence, to the development of the pathological process and to the onset of dementia are also significant [11]. According to Smits, Deeg, and Schmad [16], the potential elements of risk appear to be grouped into three factors: lifestyle, psychologically important events, and socio-environmental elements. A recent study has identified specific demographic and lifestyle behavioral data as potential risk factors for dementia, particularly in mid-life and later adulthood. The study, conducted by Li et al. in 2018 [17], found that widowhood, lower BMI, and sleep disorders were associated with an increased risk of dementia. Concurrently, an extensive review of the extant literature reveals a dearth of empirical evidence elucidating the manner through which specific lifestyle choices may precipitate the onset of cognitive impairments that could potentially initiate a diagnostic trajectory leading to dementia. Furthermore, there is a paucity of data concerning the extent to which such impairments may manifest as normal frailty, which is not considered pathological within the context of the age group in question. In view of the aforementioned findings, it can be deduced that the development of psychological strategies aimed at maintaining self-integrity in the face of highly emotionally significant events, the continuous cultivation of beneficial relational networks to support daily life, and the engagement in daily activities that have sustained a wide range of cognitive and motor functions have not exerted a discernible influence on the subsequent onset of a possible cognitive decline.

The extant literature in this field has frequently introduced the concept of cognitive reserve, which is, above all, defined as the individual differences in the ability to cope with dementia [18]. The present concept endeavors to elucidate how an individual may possess resources at the time of diagnosis that facilitate a more favorable trajectory of disease progression. Furthermore, the objective is to ascertain whether the cognitive reserve that individuals have cultivated throughout their lives could function as a form of protection against pathological cognitive decline, which could serve as an initial step toward dementia. As demonstrated in the research by Dekhtyar et al. [19], educational attainment and occupational characteristics have been identified as components of cognitive reserve, which appears to offer protection against dementia.

A mounting body of research underscores the significance of protective lifestyle factors in diminishing the likelihood of developing dementia and fostering cognitive well-being in later life. Research has demonstrated a correlation between a healthy lifestyle—comprising nutrition, physical activity, and social engagement—and a reduced incidence of cognitive decline [20] and an enhanced quality of life during the aging process [21]. Furthermore, psychological resilience, particularly the ability to cope with adverse life events (e.g., bereavement or illness), has been identified as a key factor in mitigating depressive symptoms and sustaining well-being in older adults [22,23]. The role of social networks is equally significant. These networks provide emotional support and shared experiences, thereby fostering psychological well-being in later life [24].

Despite these insights, the extant literature remains fragmented, with most studies examining individual lifestyle components in isolation rather than offering a holistic understanding of how these factors collectively contribute to dementia prevention [25]. A comprehensive approach is necessary to elucidate the interplay between lifestyle, resilience, and social engagement in preserving cognitive health [26].

In accordance with the life course perspective on the onset of dementia, the present study adopts the concept of “longevity as responsibility” [1], proposing that successful aging is contingent upon the following three fundamental pillars:

1. Optimal lifestyle habits (e.g., healthy diet, regular physical activity, effective leisure management);
2. Psychological resilience exhibited in the face of adversity;
3. Active social engagement within familiar and community networks.

The objective of this study is to integrate these domains in order to identify the daily determinants of cognitive well-being in aging. The ultimate goal of this study is to mitigate age-related frailty and dementia risk.

## 2. Methods

To gain insight into how individuals have conducted their lives and to relate this aspect to the onset of cognitive decline, a specifically tailored retrospective interview was conducted in a group of individuals who requested a neuropsychological evaluation at the Northern Italy Dementia Assessment Service (CDCD). The data obtained from the interviews were analyzed in conjunction with the results of the Mini Mental State Examination generally utilized in Italian public health services to ascertain the onset of dementia. The subsequent section will present the results of the aforementioned data collection and comparison.

### 2.1. Participants

A total of 39 individuals were invited to participate in the study during their preliminary colloquium with the primary care physician. The physician requested a subsequent neuropsychological evaluation for the potential presence of dementia. Thirty-two subjects provided informed consent to participate in the proposed research study and underwent a cognitive function assessment. Seven individuals indicated that they lacked sufficient additional time to spend with the neuropsychologist. The participants, who had volunteered to take part in the research and had signed an informed consent form for the interview, ranged in age from 61 to 83 years old (mean = 71.16; standard deviation [SD] = 7.00) and from 2 to 19 years old (mean = 6.84; SD = 4.29). Participants were selected randomly from a population of 32 individuals, with 16 females and 16 males. The subjects resided primarily in Milan, Bergamo, and other regions within Lombardy at the time of the interview. At the time of the interview, four of the participants were residing in a nursing home.

### 2.2. Interview

In order to investigate to what extent some components of the past and current lifestyle adopted by the person may have influenced the appearance of dementia the interview focused on the following topics:

- Lifestyle: Participants were requested to provide a detailed account of their past and present habits and routines in relation to their daily living. In this section of the interview, the following areas were explored:
  ○ Which of the following best describes the individual’s primary occupation for the majority of their working life?
  ○ What is the diet they have been maintained for most of their lives?
  ○ If they smoke, have smoked in the past and/or could consider themselves as a regular smoker;
  ○ What are the hobbies and/or occupations that have ordinary characterized their daily life?
  ○ Which kind of physical activity they have usually performed during lifespan?
  ○ What skills with the use of new technologies have they acquired over the course of their lives?
- Stressors: The individuals were requested to identify and describe any emotionally distressing experiences they had encountered during their lifetime, and to provide a detailed account of these experiences during the course of the interview. Specific inquiries were posed regarding the following:
  ○ If they had to face a transfer that led them to radically change their lifestyle habits;
  ○ What kind of serious illness have they had to face in their life;
  ○ How was grieving for the death of a relative, if there was one in their life;
  ○ Whether there have been particularly difficult challenges to face with in the past or recently.
- Sociability: The awareness of being part of a group of persons that has mutually engaged each other. Sociability in older age was considered as linked with spouse and family, neighborly relationships and friends’ proximity. In particular, the interview investigated the following:
  ○ How many children the persons have had and whether they currently reside in the vicinity;
  ○ If the job activity can be considered as a team-work, whatever the person is still working in;
  ○ What kind of social network can be identified in their daily activities;
  ○ How they consider their loneliness feeling, if any.

### 2.3. Procedure

In the context of a medical study centered on cognitive frailty, subjects were invited to participate in the research by their primary care physician, contingent upon the presentation of specific indications of cognitive frailty during a clinical colloquium. The subjects possess the prerogative to either accept or decline the incentive. Pursuant to the determination that had been made, the parties concerned were accordingly presented to the neuropsychologist.

The neuropsychologist provided the participants with information regarding the procedure and the informed consent form, which outlined the terms of their participation in the study. Furthermore, respondents were prompted to abstain from responding to inquiries that they regarded as excessively personal in nature.

The cognitive evaluation and interview each required approximately 30 min, with the remainder of the time dedicated to other activities. All interviews were conducted at the same medical facility where the cognitive evaluations were performed. For each participant, data were collected from the same neuropsychologist who was responsible for conducting the clinical evaluation.

Two female neuropsychologists participated in the research as interviewers. Each of the two neuropsychologists conducted the cognitive evaluation using the Mini-Mental State Examination (MMSE) and collected the interviews with half of the participants. At the time of the interview, the participants were not informed their cognitive abilities had been evaluated. A semi-structured interview was conducted and then recorded for later analysis. Subsequently, the data were transcribed in full for analysis. It was the responsibility of each neuropsychologist to transcribe the interviews conducted by the other and to provide a summary of the data collected.

### 2.4. Data Reduction and Analysis

MMSE score was calculated according to the standard norms and the raw score obtained was adjusted for individual age and educational levels according to the score standardization for the Italian population.

According to the criterion specified beside each factor, the following factors were taken into consideration to determine a 0–1 score from the transcript:

- Lifestyle: *Educational Level* (1 for >7 years; 0 for ≤7 years); *Work Activity* (1 for organizational type; 0 for manual type); *Driving License* (1 for still active; 0 for expired); *Hobby* (1 for having one different form the job activity; 0 for not having or having similar to the job activity); *Diet* (1 for balanced according to nutritional standards; 0 for unbalanced); *Smoke* (1 for not smoking or having stopped to smoke from ≥30 years; 0 for smoking); *Technology* (1 for using of smartphone computer or tablet; 0 for not using one). *Physical activity* (1 for having had a regular physical activity; 0 for not having had one). The X_1_ variable range calculated accordingly is 0–8.
- Stressors: *Wedlock* (1 for being widower; 0 for being already married); *Work Retirement* (1 for being retired; 0 for being already not retired); *Home Moving* (1 for moving occurred in an epoch corresponding to 1/3 of their life; 0 for not having had home moving, or occurred less for 1/3 of the entire individual lifespan); *Illness* (1 for having had serious illness; 0 for not having had one); *Rare event* (1 for having faced an uncommon event; 0 for not having faced one); *Abandonment* (1 for have been left from a relative; 0 for not have experienced abandonment). The X_2_ variable range calculated accordingly is 0–6.
- Sociability: *Marriage* (1 for being already married; 0 for being widower); *Children proximity* (1 for having a son/daughter close to them; 0 for having son/daughter far from them); *Work Relationships* (1 for being already not retired; 0 for being retired); *Travels* (1 for usually participating in travel for pleasure; 0 for occasionally or not participating); *Friendships* (1 for usually having activity with friends; 0 for not having it); *Loneliness* (1 for not having experienced the condition of being secluded; 0 for having it). The X_3_ variable range calculated accordingly is 0–6.

The response variable MMSE is modelled as the numerical variable to apply an ordinary linear regression model.

Due to the limited number of cases, a first a preliminary data exploration was carried out by applying a univariate linear regression model to all the factor as covariates. The estimated parameter (EST) and the *p*-value (*p*) are collected in Table 1.

Gender factor was not included due to the non-significant value provided from the t-test (Male MMSE Mean = 21.79, Female MMSE Mean = 21.82, t-statistics −0.02, *p*-value 0.98). Following the suggestion of the result of this first analysis we propose two multivariate linear regression models. The first one models the MMSE score as the function of the three aggregate variables described previously: X_1_ Lifestyle, X_2_ Stressors and X_3_ Sociability. A stepwise procedure selects a model with only the covariate Lifestyle. The estimated parameter (EST) the standard error (SD), the *p*-value (*p*) and the R^2^ statistics (R^2^) for the univariate linear model are collected in Table 2. The second one explains the MMSE score with the first 4 significative factor according to the preliminary analysis.

The estimated parameter (EST) the standard error (SD the *p*-value (*p*), the multiple R^2^ statistics (MR2) and the adjusted R^2^ statistics (AR2) are collected in Table 3. A stepwise procedure also selects the best model for this multivariate regression model.

## 3. Results

The different colors used in Table 1 identify in which aggregate variable the single factor is considered. Factors concerning the Lifestyle are mainly depicted in the top of the Table 1 (corresponding to higher significance, *p* < 0.05) while factors concerning Stressor tend to appear in bottom part of the Table 1 (corresponding to lower significance). The factors concerning Sociability are more spread in Table 1. In particular within Sociability, the Friendship factor appears as very significative (*p* = 0.01). The results on Table 2 confirm that the aggregate variable Lifestyle better explain the MMSE score (*p* = 0.004). The step wise procedure applied to the multivariate linear model with the three aggregate variables select the model with only the Lifestyle variable (first line of Table 2).

The second multivariate linear model considers as an explanatory variable the significative factors (*p*-value < 0.05). The results of Table 1 suggest a model with Technology, Driving License, Friendship and Educational Level. The results in Table 3 show that the first two factors are significative at 5%, the Friendship is significative at 10% and the Educational level is not significative. The adjusted R^2^ for this complete model is 0.49.

The stepwise procedure selects the model with the first three variables. Comparing the result of Table 3 and Table 4 we can see how the all the value of the Estimate parameter increase with a decreasing of the SE, this mean that the estimates are more precise. Moreover, the adjusted R^2^ has a little increase.

**Figure 1:**
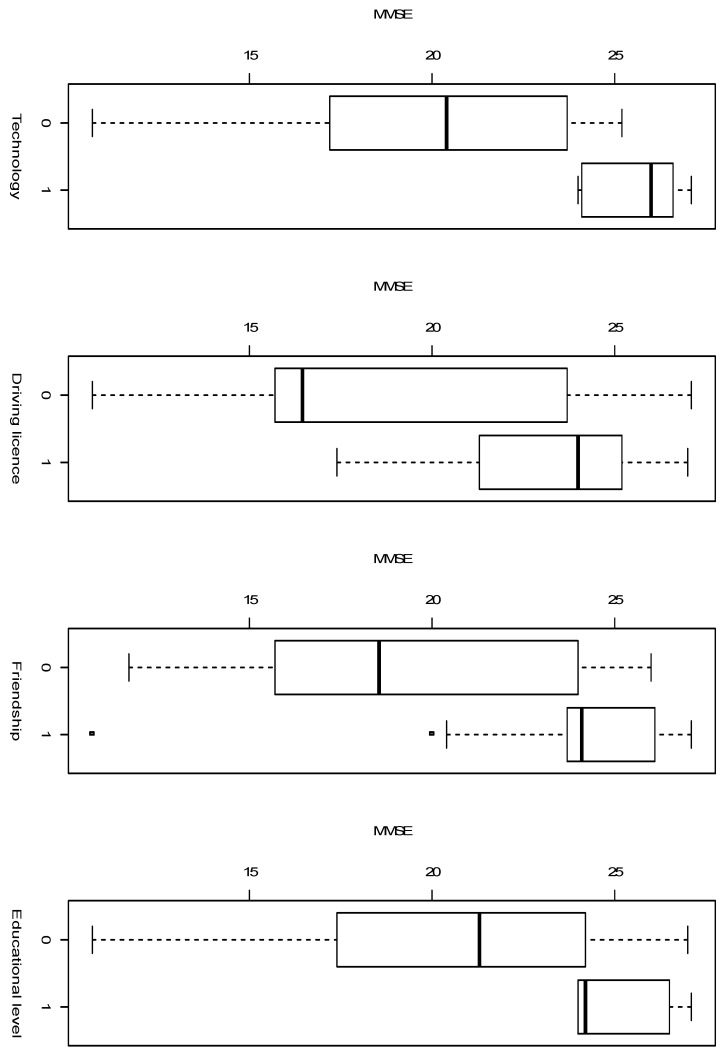
Boxplot of MMSE according to the factor: Technology, Driving Licence, Friendship and Educational level.

The positive and high value of the parameters suggest that these three factors are important to predict the value of the MMSE score. The value of the MMSE score with respect to the four factors are described through the boxplots in Figure 1.

What it is evident in the boxplots, is the higher variability of the MMSE score when the factor takes value 0, with respect to a higher median value and a lower variability when the factor takes value 1.

## 4. Discussion

The results appear to suggest that daily experiences throughout the lifespan have exerted a multifaceted influence on the onset of cognitive frailty during the aging process. Specifically, beyond educational attainment, particular activities, including independent driving and technological literacy, in conjunction with sustaining a social network, exhibited a substantial influence on performance on a cognitive screening test for dementia, such as the MMSE.

The preliminary data exploration revealed that the majority of factors pertaining to lifestyle exhibited the highest significance in predicting a favorable level of MMSE. Among the lifestyle factors, the greatest protective effect against cognitive decline was observed for those who used technology extensively. This finding aligns with the conclusions of research conducted by Cody et al. [27] and Benge [28], which demonstrated that spending a limited amount of time in front of a computer can mitigate the cognitive decline associated with aging. The integration of computers and other digital devices into later life fosters a sense of contemporary engagement, thereby facilitating a perception of technological advancement alignment. Additionally, the acquisition of internet navigation skills has been demonstrated to enhance an individual’s sense of self-worth and confidence. Concurrently, the expansion of the scope of cognitive stimulation and the complexity of the information to be processed during online searches can facilitate cognitive training in adulthood and in older age. Conversely, Niggard and Kottorp [29] posited that individuals diagnosed with Mild Cognitive Impairment perceive the utilization of technologies as a significant obstacle and as a non-integrated aspect of their daily lives. Therefore, from a protective standpoint, the stimulation of multitasking cognitive efforts throughout the lifespan can ensure the maintenance of adequate cognitive flexibility. This, in turn, can contribute to the development of cognitive reserve in aging. Alternatively, if cognitive decline is already present at this stage of life, the introduction of new technology-based tasks may serve to exacerbate an existing condition.

The other significant predictor of a favorable cognitive outcome on the MMSE was the ability to operate a motor vehicle independently, as indicated by the *Driving License* factor. While there is an absence of existing literature that explicitly demonstrates a direct correlation between driving proficiency and the prevention of cognitive decline, research has indicated that individuals with extensive driving experience exhibit a distinct organization of attention and spatial memory compared to those without cognitive impairment. Indeed, data obtained from taxi drivers in London [30] has indicated that spatial knowledge derived from driving may be associated with specific patterns of hippocampal functioning. The intricate geographic configurations of a city appear to reinforce memory and facilitate reasoning about street configurations for individuals tasked with memorizing complex pathways [31]. Driving in an urban environment is known to require the exertion of more intricate cognitive processes. These processes are necessary to navigate the myriads of daily challenges that arise throughout the lifespan. Therefore, the acquisition of a driver’s license may serve as an indicator of consistent cognitive performance over an extended period. Several studies have consistently reported a modification of gamma oscillations in the entorhinal-hippocampal circuit in patients diagnosed with Alzheimer’s dementia [32,33]. Furthermore, numerous datasets have been published concerning the progressive plasticity of the hippocampus in the context of exploring complex environments in the setting of aging [34,35]. It can be posited that driving ability may be considered a means of enhancing the likelihood of actively exploring an enriched environment. Furthermore, the act of navigating a driving environment presents a unique opportunity for motor and cognitive choices, such as performing driving behaviors and following turn types. This may, in turn, prove beneficial in the prevention of neurodegeneration in the hippocampus and the deceleration of cognitive frailty in aging. In summary, it can be concluded that driving ability exerts a positive influence on the mitigation of neurodegeneration in the hippocampus and the deceleration of cognitive decline during the aging process.

A thorough examination of the data revealed that the *Friendship* factor emerged as a particularly salient aspect of sociability, while the other factors exhibited a more dispersed pattern. The data indicate the pivotal role of relationships and social networks in the life of every individual. Individuals with a limited number of relationships and a suboptimal quality of relationships appear to demonstrate a significantly elevated risk of developing neurodegenerative disease, as indicated by MMSE-related values. In accordance with the findings of our study, a research investigation [36,37] examined the possible association between dementia and social isolation. A comprehensive literature review of approximately twenty longitudinal cohort studies identified an association between dementia and a low level of social participation, sporadic social contact, and a high level of meaningful interactions. Contrary to the results of our study, the present investigation did not identify a significant correlation between satisfaction with one’s social network and the onset of dementia [38]. Furthermore, the potential impact on cognitive function of living in solitude and without social interaction should be considered. This is largely attributed to the fact that such individuals are no longer exposed to these functioning on a regular basis. A longitudinal study was conducted to examine the health and well-being of over 2000 individuals without any indications of dementia or cognitive impairment who resided in a confinement condition for about three years. The findings revealed the emergence of dementia-related symptoms in those who lived alone. In multiple cognitive assessments, approximately one in ten individuals who reported feelings of loneliness showed cognitive decline, while one in twenty individuals who lived with others showed potential signs of the onset of dementia [39]. It is important to stress that loneliness can have two different effects. On the one hand, it can be an indicator of developing dementia. On the other hand, it can manifest itself as a behavioral response to cognitive impairment. A study of socially isolated individuals suggests that participation in cultural events, volunteering and musical activities such as singing in a choir can improve longevity and quality of life [40,41]. Although, in the first regression model, *Educational Level* appears to be a factor that could have influenced the MMSE score in our subjects, in a second regression model its role in protecting against cognitive decline appears to be less influential. These data seem to be in contrast with studies that have shown how a high level of schooling increases the level of cognitive reserve that enables a protective effect against the development of dementia and AD [42,43]. However, when interpreting the existing literature on the subject, it is important to recognize the prevailing view that people with higher levels of education are more likely to enjoy greater economic prosperity, a higher quality of life and better access to health care. As a result, they are better protected against cardiovascular disease and stroke, both of which contribute to the development of dementia. Some scholars [44] have also suggested that people with low levels of education may simply have greater difficulty performing cognitive tests, and therefore the apparent association between education and dementia would be a measurement artefact. In this regard, Meng and collaborators [45] have observed that approximately 15–30% of individuals with mild and moderate brain pathology at the time of death have no manifest clinical symptoms in late life. This would support the hypothesis that some people may be able to counterbalance for neuropathology by preventing and delaying cognitive symptoms that are generally identified by MMSE. For all these reasons, the data we found on educational level might not have been t significant to a more in-depth analysis such as a stepwise multivariate regression model.

Finally, the factors addressable as stressors correspond to a lower prediction significance in preliminary analysis with the higher value (even if not significant ones) for *Wedlock*, *Rare Event*, and *Abandonement.* Although the occurrence of stressful life events has often been referred to as one of the suggested psychosocial risk factors for dementia, as the experience of negative events can cause stress reactions, this finding does not seem to be confirmed by our results. Clemens’s [46] hypothesis posits that events with a high negative emotional impact may influence the complex neuroendocrine process regulated by the hypothalamic-hypophysis-adrenal axis. This process, in the presence of a potent stressor, has been observed to induce an excess of cortisol in certain neurons, particularly those located in the hippocampus. According to this study there are four risk factors that are found to be significantly higher: the death of a close relative, hard work during adulthood, the diagnosis of a disabling illness, and the loss of a child. In front of these results, a cumulative incidence of these factors could be noted: starting from a percentage of 3.3% in non-exposed subjects, to 8% in subjects with one or two risk factors to reach 19% in subjects with three or more factors. In our study this data does not appear to be confirmed, even if several individuals reported life events that can be defined as high level stressors. One possible interpretation of these data could be to consider psychological resilience, defined as the ability to achieve good environmental functioning and develop individual potentialities in the context of a life full of severe adversities [47]. Being resilient therefore means having the strength to respond to traumatic or stressful events and to reorganize your life in a positive way. The American Psychological Association has identified among the key points to positively plan one’s future in a resilient way is the ability to maintain good relationships with close family members, with friends, and with those whom the individual considers important, in order to create a social network in response to crises and to the difficulties of life [48]. The hypothesis that the support and assistance provided by one’s own social environment is a primary factor in the development of growth, resilience, and adaptability seems to be supported by the findings of the interviews conducted. In our case, having an active network of friends has emerged as a key factor in mitigating the negative effects of life’s challenges on a person’s lifelong development.

In summary, the results were also confirmed by the multivariate linear model with the three aggregate variables, in which lifestyle appeared to better explain the MMSE score. While sociability, as an aggregate, failed to show a significant value for the MMSE, the corresponding aggregate factors of stressors had no predictive value for possible cognitive decline.

An interesting aspect of the relationship between lifestyles and the risk of dementia is its potential impact on prevention. As lifestyles are modifiable, they are the typical focus of information programs and health education. Furthermore, the interconnectedness of healthy lifestyles is evident in how they interact with each other. For example, cigarette smoking is associated with alcohol consumption, and vice versa. Additionally, low levels of physical activity are related to high-calorie, unbalanced diets. The risk of social withdrawal increases with lower levels of well-being. Consequently, it is difficult to determine the potential health effects of a specific lifestyle. For this reason, many argue that healthy lifestyles must be studied in clusters rather than separately [49,50]. Accordingly, in our data, the estimated values on MMSE score for Lifestyle as an aggregate factor for each of *Technology*, *Driving License* and *Friendship* suggests that it is possible to intervene in an integrated way for cognitive decline prevention. In fact, all of them showed a positive effect on maintaining high cognitive function in people over 65. Therefore, modifying at least one healthy habit (e.g., quitting smoking or adopting a balanced diet) appears to improve the MMSE score by more than one point. Similarly, modifying social habits (e.g., cultivating a more robust friendship network), maintaining driving privileges (e.g., autonomous movement in surroundings), and utilizing new technologies (e.g., web browsing or communication with friends) can improve the MMSE score by more than six points. In situations that are not clearly defined, such as the Mini-Mental Status Examination (MMSE) cut-off score for potential dementia (less than 24), the significance of this value is paramount. Adopting protective habits early in life can influence this score, thereby demonstrating the potential impact of proactive lifestyle modifications on long-term health outcomes.

In summary, the findings of this study indicate that factors other than nutrition, occupational history, stressful life events, and hobbies are associated with successful aging and the prevention of dementia. Specifically, the analysis suggests a correlation between maintaining an independent lifestyle in a technologically advanced environment and the ability to share this way of life with friends. The present study’s findings suggest that the aforementioned way of life, which was described by the study’s participants and revealed to be indicative of optimal cognitive functioning in late life, may serve as a valuable source of information for the development of long-life educational programs. These programs, if implemented during early adulthood, have the potential to safeguard future generations from cognitive frailty and dementia.

However, these findings must be interpreted with caution, as the small sample size (N = 32) represents a significant limitation of the study. The exploratory nature of the analysis, combined with limited statistical power, may increase the risk of both type I (false positive) and type II (false negative) errors. As such, while some associations reached statistical significance, they cannot be generalized beyond the specific study population and should be considered preliminary until replicated in larger, more representative samples.

## Figures and Tables

**Table 1:** The estimated value and the *p*-value for any model each with one covariate taking value 0 or 1 as explained in Data Reduction. Lifestyle factors are depicted in light blue, stressors in yellow and sociability in green. Educational level is considered as transversal through the factors.

Factor	EST	*p*
Technology	5.54	0.00
Driving Licence	5.07	0.00
Friendship	3.94	0.01
Educational Level	4.33	0.02
Work Activity	3.36	0.10
Physical Activity	2.54	0.11
Smoke	−2.64	0.20
Work Relationships	3.05	0.21
Marriage	2.13	0.22
Wedlock	−2.36	0.22
Rare Event	1.90	0.26
Abandonment	−1.09	0.51
Loneliness	1.09	0.51
Children	−1.10	0.56
Hobby	−0.72	0.66
Diet	0.58	0.73
Work Retirement	0.66	0.74
Illness	0.55	0.74
Travel	−0.36	0.84
Home Moving	0.10	0.96

**Table 2:** The result for the three linear models, each with only one of the aggregate variables.

Variable	EST	SE	*p*	R^2^
Lifestyle	1.25	0.40	0.004	0.24
Stressors	0.01	0.60	0.981	0.00
Sociability	0.83	0.53	0.130	0.07

**Table 3:** The result for the multiple linear model with the 4 significative covariates according to the preliminary analysis.

Variable	EST	SE	*p*	
Technology	3.41	1.53	0.035	
Driving Licence	3.13	1.31	0.025	
Friendship	2.38	1.19	0.056	0.55 (MR2)
Educational Level	0.95	1.67	0.577	0.49 (AR2)

**Table 4:** The result for the multiple linear model with the 3 significative covariates selected by the stepwise procedure.

Variable	EST	SE	*p*	
Technology	3.88	1.28	0.005	
Driving Licence	3.12	1.30	0.023	0.55 (MR2)
Friendship	2.41	1.17	0.056	0.50 (AR2)

## Data Availability

The data presented in this study are available on request from the corresponding author due to ethical reasons.
